# The association of antidepressant drug usage with cognitive impairment or dementia, including Alzheimer disease: A systematic review and meta‐analysis

**DOI:** 10.1002/da.22584

**Published:** 2016-12-28

**Authors:** John Moraros, Chijioke Nwankwo, Scott B. Patten, Darrell D. Mousseau

**Affiliations:** ^1^ School of Public HealthUniversity of SaskatchewanSaskatoonSKCanada; ^2^ Departments of Community Health Sciences and PsychiatryUniversity of CalgaryCalgaryABCanada; ^3^ Cell Signalling LaboratoryDepartments of Psychiatry and PhysiologyUniversity of SaskatchewanSaskatoonSKCanada

**Keywords:** antidepressants, cognition, depression, geriatric/aging/elderly, pharmacoepidemiology

## Abstract

**Objective:**

To determine if antidepressant drug usage is associated with cognitive impairment or dementia, including Alzheimer disease (AD).

**Method:**

We conducted a systematic search of Medline, PubMed, PsycINFO, Web of Science, Embase, CINAHL, and the Cochrane Library. An initial screen by abstracts and titles was performed, and relevant full articles were then reviewed and assessed for their methodologic quality. Crude effect estimates were extracted from the included articles and a pooled estimate was obtained using a random effects model.

**Results:**

Five articles were selected from an initial pool of 4,123 articles. Use of antidepressant drugs was associated with a significant twofold increase in the odds of some form of cognitive impairment or dementia (OR = 2.17). Age was identified as a likely modifier of the association between antidepressant use and some form of cognitive impairment or AD/dementia. Studies that included participants with an average age equal to or greater than 65 years showed an increased odds of some form of cognitive impairment with antidepressant drug usage (OR = 1.65), whereas those with participants less than age 65 revealed an even stronger association (OR = 3.25).

**Conclusions:**

Antidepressant drug usage is associated with AD/dementia and this is particularly evident if usage begins before age 65. This association may arise due to confounding by depression or depression severity. However, biological mechanisms potentially linking antidepressant exposure to dementia have been described, so an etiological effect of antidepressants is possible. With this confirmation that an association exists, clarification of underlying etiologic pathways requires urgent attention.

**Abbreviations**
AD: Alzheimer disease
MAO: monoamine oxidase
SNRIs: serotonin noradrenaline reuptake inhibitors
SSRIs: selective serotonin reuptake inhibitors
QUIPS: Quality of Prognosis Studies in Systematic Reviews

## 1 INTRODUCTION

Our increasing lifespan has led to an increased prevalence of diseases of the brain, including Alzheimer disease (AD), a progressive, neurodegenerative disorder with significant cognitive and behavioral deficits, which accounts for almost 80% of cases of dementia (World Alzheimer Report, 2014; World Health Organization, 2015a). In rare cases, AD is associated with autosomal dominant mutations in specific genes that enhance amyloid burden in the brain; however, the majority of cases are of unknown origin and are termed late‐onset given that symptomatology manifests in older individuals (Tanzi, 1999). The prevalence of AD at 65 years of age is 13% and doubles every 5 years thereafter (World Alzheimer Report, 2014). The World Health Organization estimates that globally there are 47.5 million cases of AD/dementia (Aguero‐Torres et al., 1998) that annually incur costs surpassing $604 billion USD (World Alzheimer Report, 2014). The economic and social burden of AD/dementia is expected to triple by 2050 (World Health Organization, 2015a).

Significant efforts are being made to identify modifiable risk factors for AD, specifically any factor that influences the earliest stages of the disease process, when intervention would still provide therapeutic benefit. Although the causes of AD are not yet fully understood, it is believed that late‐onset AD results from an interaction of genetic (i.e. *APOE* ε4 status), lifestyle, and environmental risk factors (Glenner & Wong, 1984; Poirier et al., 1993; Tanzi, 2012).

Age and sex remain two of the primary risk factors for AD (Richard et al., 2012). Yet, neither a Canadian Study of Health and Aging report (Lindsay et al., 2002) nor the Framingham study (Bachman et al., 1993) found any sex‐dependent prevalence in AD. If one considers that there is a similar prevalence in males and females in the early stages of AD, but a strong female prevalence in severe cases, then this could be interpreted to suggest that males might die sooner after their AD becomes severe (Aguero‐Torres et al., 1998; Hy & Keller, 2000). In support of this, a previous study of ours based on provincial (Saskatchewan, Canada) health care utilization data found a higher risk of mortality in demented male patients with a comorbid psychiatric disorder when compared with demented patients (either male or female) with no psychiatric history (Meng et al., 2012).

Depression is now acknowledged as a risk factor for AD/dementia (Katon et al., 2012; World Health Organization, 2015b). It has been proposed as one of the neuropsychiatric disorders that is a marker (Ismail et al., 2016), or potentially a prodrome (Schweitzer, Tuckwell, O'Brien, & Ames, 2002; World Alzheimer Report, 2014), for incident AD/dementia in certain cohorts, and can alter the risk for AD as much as twofold (Caraci, Copani, & icoletti, 2010; Geerlings et al., 2008; Masters, Morris, & Roe, 2015; Wuwongse, Chang, & Law, 2010), even if the diagnosis of depression is made 17 years (i.e. the Framingham study) (Saczynski et al., 2010) or 25 years (i.e. the MIRAGE study) (Green et al., 2003) prior to the onset of AD.

Depression is one of the most common mental health conditions globally (Collins et al., 2011; World Health Organization, 2015b) and the prescription of antidepressant drugs, particularly the selective serotonin reuptake inhibitors (SSRIs), has increased dramatically over the last three decades (Pratt, Brody, & Gu, 2011) with almost half of the prescriptions being for an off‐label indication (e.g. anxiety, insomnia and pain (Wong et al., 2016). Several studies (Chen et al., 2013; Han et al., 2011; Herrera‐Guzman et al., 2010; Jorge et al, 2010; Nair et al., 2014; Rozzini et al., 2010) have shown behavioral and cognitive improvement associated with antidepressant drug usage in patients with a range of neurologic and psychiatric diagnoses, although the literature also provides instances that might question any beneficial effect of antidepressant drug usage in cognitive decline (Ardal & Hammar, 2011; Dawes et al., 2012; Kessing, Forman, & Andersen, 2011; Rosenberg et al., 2012). The possibility that these drugs might not benefit all patient populations and actually could be contributing to risk of iatrogenic cognitive decline (i.e. AD/dementia) in a vulnerable cohort could help explain some of the heterogeneity in the etiology, age of onset, and/or rate of disease progression in AD. To the best of our knowledge, there are few studies that have shown an association between antidepressant drug usage on AD/dementia. This may be due to a lack of adequate precision/power in those studies. We conducted a systematic review and meta‐analysis to address this gap.

## 2 METHODS

### 2.1 Data sources

**Figure 1:**
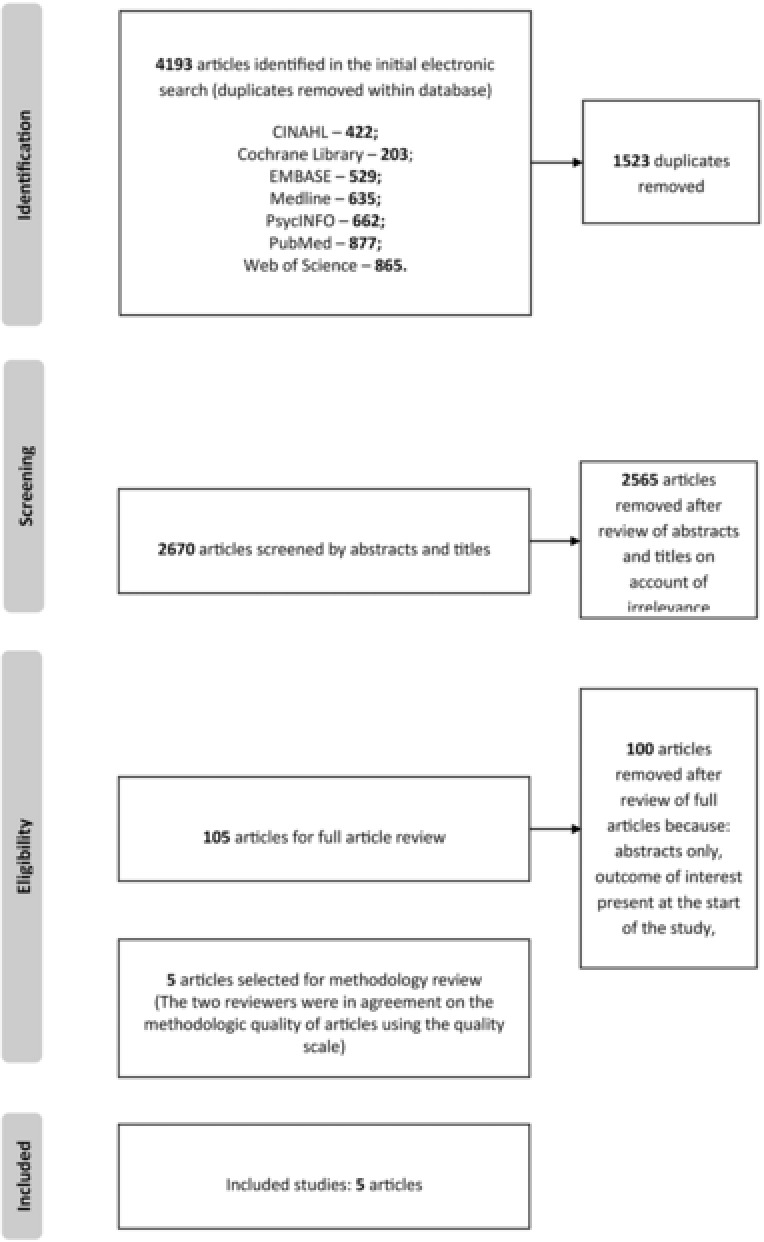
Flow diagram for included studies

We conducted a search for peer‐reviewed articles across databases such as Medline, PubMed, PsycINFO, Web of science, Embase, CINAHL, Cochrane library. Gray literature search was carried out using google scholar. Searches were carried out with a combination of the following key words: Antidepressant, antidepressive agent, thymoleptic, depression medication, depression therapy, depression treatment, monoamine oxidase inhibitors, SSRIs, tricyclic antidepressant, AD, dementia, demented, cognitive impairment, cognitive loss, cognitive deficit (see Fig. 1 for search strategy).

The identification of eligible studies began with an examination of titles and abstracts to remove duplicates. Second, two of the authors (CN and JM) reviewed the full‐text articles for relevance and agreement guided by predetermined inclusion and exclusion criteria. Third, methodologic quality was assessed by two reviewers (CN and JM) independently using the Quality of Prognosis Studies in Systematic Reviews (QUIPS) tool (Hayden, Cote, & Bombardier, 2006). There was full agreement between the two reviewers on which studies to include and the interrater variability was high (Cohen's kappa 0.88). If any disagreement was encountered, this was resolved by consensus‐seeking discussion.

### 2.2 Study selection

An article was included if it met the following inclusion criteria: (a) it was published in the English language, (b) full text was available, (c) it had a comparative design with two groups; one being exposed to antidepressant drug use and the other group being antidepressant naïve (as reported), (d) it was not older than 10 years, and (e) it had quantifiable odds ratios describing the effect size. Articles were excluded from analysis if: (a) it described a study with a single arm or without a comparison group, and/or (b) it did not describe a clear categorization of the outcome as dementia or cognitive impairment.

### 2.3 Data extraction

Odds ratios and their associated 95% confidence intervals (CIs) were obtained (if given) or calculated (when not directly available) from the articles. Other relevant information extracted from each study included: the study design, presence of depression at baseline, specific type of outcome (dementia vs cognitive impairment), predominant sex of study participants, average age of study participants and the use of other psychotropic medications.

### 2.4 Data analysis

The outcome of interest was the occurrence of dementia or any other cognitive disorder. Use of antidepressant drugs was the primary predictor of interest. The natural log of the odds ratios and the natural log of their associated 95% confidence intervals were computed for each study. The included studies were categorized for subgroup analysis based on the study duration into studies greater than or equal to 10 years and those less than 10 years. Categorizations were also made based on the average age of the included participants in the studies using a cut‐off point of 65 years of age.

Given the nature of the research question and the likelihood of clinical heterogeneity, a random effects model defined by the equation *Y*_i_ = *μ* + *u*_i_ + *ε*_i_ *_,_* was used to obtain a pooled estimate of the effect (where *μ =* the average effect, *u*_i_ = variation from the average effect, and *ε*_i_ = residual error). This allowed for an estimation of the amount of variation both within and between studies. We assumed a logit link function for binary outcome.

Statistical analysis for heterogeneity was carried out using Higgins I‐squared (Higgins & Thompson, 2002) and further explored with the use of subgroup analysis based on categorizations determined a priori. The robustness of the findings was assessed by determining the influence of each individual study on the overall pooled estimate using Tobias’ method (1999). This involved a reestimation of the pooled estimate with each study omitted one after the other. Publication bias was graphically assessed with the use of a funnel plot and statistically by calculating Begg's test (Begg & Mazumdar, 1994). All analyses were performed with STATA version 13.1 using the “Metan” command with statistical significance determined by *P* values less than 5%.

## 3 RESULTS

### 3.1 Study selection

The initial search parameters identified 4,193 articles. Of these, 1,523 were removed as duplicates. The rest of the studies were carefully screened by title and abstract, and an additional 2,565 were removed for not being relevant to our study. This eventually yielded a pool of 105 articles; the full articles were then retrieved and further screened for relevance. Five of the articles were deemed relevant by both reviewers after the full article screening. These five articles underwent methodology quality review from which all five articles passed and were included in the meta‐analysis (Fig. 1).

### 3.2 Methodological quality

The methodological quality review of the studies is reported in Table 1. The main methodology deficit noted among the included studies was the absence of blinding among the assessors. Also, most of the studies did not explicitly state if any differences existed between participants included in their study and those not included. Strengths in the methodology of the included studies ranged from adequate description of the source population, adequate outcome data at follow‐up, proper assessment of outcome, avoidance of overfitting issues (i.e. at least 10 participants in the smallest group per predictor and outcome variable), proper statistical analysis, and consideration of important confounders.

**Table 1:** Methodology quality assessment

**ARTICLE**	Source population adequately described	70% have outcome data at follow‐up	No important differences between participants who completed the study and those who did not	Primary predictor is adequately measured in study participants to sufficiently limit potential bias	70% had complete data for primary predictor	Outcome assessors are blinded for the exposure status	The outcome is based on assessment performed by a multidisciplinary team using set criteria	Important potential confounders are measured	The statistical analysis is appropriate for the design of the study	There is no over fitting
Angst et al. (2007)	**X**	**X**	**−**	**−**	**−**	**−**	**X**	**X**	**X**	**X**
Cherbuin et al. (2009)	**X**	**X**	**−**	**−**	**X**	**−**	**X**	**X**	**X**	**X**
Goveas et al. (2012)	**X**	**X**	**−**	**X**	**X**	**−**	**X**	**X**	**X**	**X**
Kessing et al. (2009)	**X**	**X**	**X**	**X**	**X**	**−**	**X**	**X**	**X**	**X**
Sachdev et al. (2012)	**X**	**X**	**−**	**−**	**X**	**−**	**X**	**X**	**X**	**X**

X, reported in study; –, not reported in study.

### 3.3 Characteristics of the pool

There were 48,267 instances of dementia or cognitive disorder and a combined total of 688,237 individuals had used antidepressant drugs (Table 2). A total of 1,477,626 participants were pooled from the five included studies (see Table 3). Participants were recruited from the general population in four of the studies (Angst et al., 2007; Cherbuin et al., 2009; Goveas et al., 2012; Kessing, Sondergard, Forman, & Andersen, 2009) and from hospital admissions in the other study (Sachdev et al., 2012). One of the studies was a randomized controlled trial (RCT) (Goveas et al., 2012), one was a prospective cohort study (Cherbuin et al., 2009), two were retrospective cohort studies (Angst et al., 2007; Kessing et al., 2009), and one was a cross‐sectional study (Sachdev et al., 2012). Exposure assessment (current antidepressant use) was obtained from the participants in three of the studies (Cherbuin et al., 2009; Goveas et al., 2012; Sachdev et al., 2012) and from existing records for the other two studies (Angst et al., 2007; Kessing et al., 2009). Outcome assessment was made for all five studies on the basis of using standardized diagnostic classifications such as ICD 9, ICD 10, and DSM IV (Table 2).

**Table 2:** Participant characteristics

STUDY	Average age (yrs) of participants	Cases	Non cases	Exposed to antidepressants	Not exposed to antidepressants	Baseline status	Outcome	Predominant sex (M‐F %)	Use of other psychotropic meds
Angst et al. (2007)	66.8	88	318	132	274	Mood disorder	Dementia	Predominantly female (F 72%, M 28%)	X
Cherbuin et al. (2009)	62.5	64	2018	102	1980	Normal cognition	Any MCD	Predominantly male (F 49%, M 51%)	X
Goveas et al. (2012)	70.9	471	6527	383	6615	Mood disorder	MCI / Probable dementia	Predominantly female (F 100%, M 0%)	**−**
Kessing et al. (2009)	57.4**	47348	1420035	687552	779831	Not specified	Dementia	Predominantly female (F 57%, M 43%)	**−**
Sachdev et al. (2012)	78.5	296	461	68	689	Normal cognition	MCI	Predominantly female (F 56%, M 44%)	**−**

*Note*: Cases: Individuals with dementia, MCI, or any other cognitive disorder.

X, reported in study; –, not reported in study; **, median values reported (no overall value was reported. Thus, a cautious approach was taken by using the value for the exposed group that was higher than the value for the nonexposed group).

MCD, mild cognitive disorder; MCI, mild cognitive impairment.

**Table 3:** Study characteristics

Author, publication year, country	Study design	*N*	Crude OR (95% CI)	Study weights	Participant source	Method of outcome assessment	Method of exposure assessment
Angst et al., 2007, Switzerland	Retrospective cohort	406	1.68 (1.03– 2.73)	19.44	Hospital admissions	Global assessment scale and ICD 9	Hospital records
Cherbuin et al., 2009, Australia	Prospective cohort	2082	3.39 (1.62– 7.06)	14.73	General population	Clinical dementia rating and DSM IV	Obtained from participants
Goveas et al., 2012, USA	Prospective cohort	6998	2.12 (1.40–3.21)	20.87	General population	CERAD neuropsychological battery and DSM IV	Obtained from participants
Kessing et al., 2009, Denmark	Retrospective cohort	1467383	3.25 (3.19– 3.32)	25.89	General population	ICD 10	Medicinal product statistics
Sachdev et al., 2012, Australia	Cross sectional study	757	1.18 (0.71–1.95)	19.08	General population	DSM IV	Obtained from participants

*Note: ICD, International Classification of Diseases; DSM, Diagnostic and Statistical Manual; CERAD, Consortium to Establish a Registry for Alzheimer's Disease*.

### 3.4 Pooled analyses

**Figure 2:**
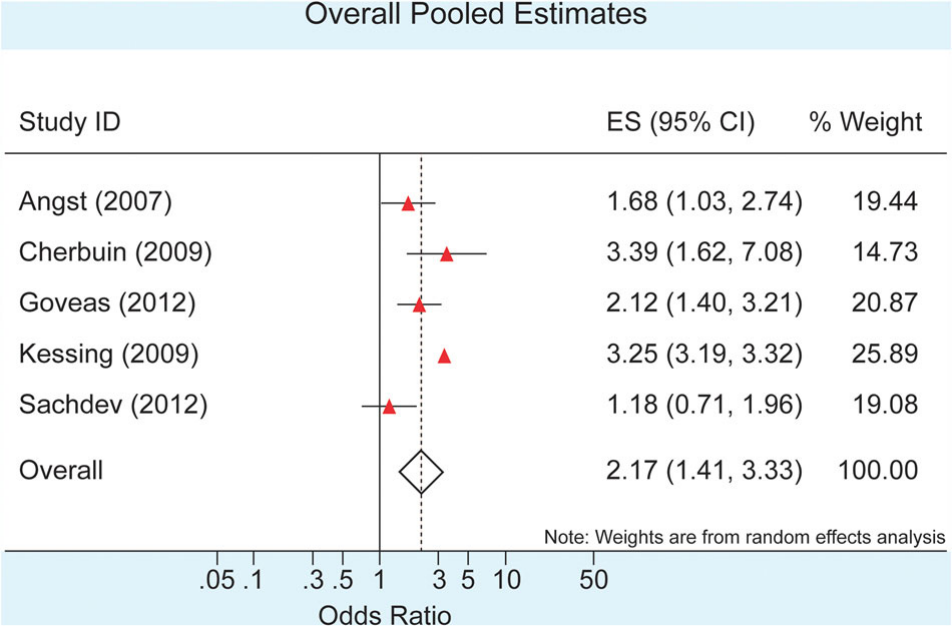
Forest plot showing estimated odds ratios and associated 95% confidence intervals (CI) for the five included studies. The pooled estimate is also shown

Overall, the pooled estimates from the five studies showed that the use of antidepressants was associated with a significant twofold increase in the odds of dementia/cognitive impairment (OR = 2.17; 95% CI: 1.41–3.33; *I^2^* = 84.9%) (Fig. 2). Approximately 85% of the variability of the estimates could be attributed to differences that existed between the studies. Subgroup analysis on factors determined a priori was carried out to further explore the potential reasons for between‐study variations. Regarding the study designs used, the only RCT included (OR = 2.12; 95% CI: 1.40‐3.21) (Goveas et al., 2012) and those with a cohort design (OR = 2.67; 95% CI: 1.71‐4.19; *I^2^* = 71.6%) (Angst et al., 2007; Cherbuin et al., 2009; Kessing et al., 2009) showed similar increments as those observed with the overall estimate. The only study that used a cross‐sectional design found no significant association between antidepressant use and some form of cognitive impairment (OR = 1.18; 95% CI: 0.71–1.96) (Sachdev et al., 2012). When considering the specific types of outcome, the studies with an outcome of dementia (Angst et al., 2007; Kessing et al., 2009) showed a pooled OR of 2.45 (95% CI: 1.29–4.64; *I^2^* = 85.8%) while those that focused on mild cognitive impairment or any other type of cognitive impairment (Cherbuin et al., 2009; Goveas et al., 2012; Sachdev et al., 2012) showed a pooled OR of 1.95 (95% CI: 1.14–3.35; *I^2^* = 67%).

Two studies involved participants with baseline depression (Angst et al., 2007; Goveas et al., 2012). The pooled OR for this group, OR = 1.92 (95% CI: 1.40–2.64; *I^2^* = 0%), was less than that for the groups without a documentation of depression at the start of the study, i.e. OR = 2.36 (95% CI: 1.19–4.67; *I^2^* = 87%); however, this difference was not statistically significant (Cherbuin et al., 2009; Goveas et al., 2012; Sachdev et al., 2012). All but one of the included studies had predominantly female participants (Angst et al., 2007; Goveas et al., 2012; Kessing et al., 2009; Sachdev et al., 2012). Grouping by this factor yielded a pooled effect of OR = 2.00 (95% CI: 1.21–3.29; *I^2^* = 88.7%) for the studies with predominant female participation. The single study (Cherbuin et al., 2009) which had a slight predominance of male participants had an OR = 3.39 (95% CI: 1.62–7.07). Studies that included participants with an average age greater or equal to 65 years old (Angst et al., 2007; Goveas et al., 2012; Sachdev et al., 2012) showed an increased odds of some form of cognitive impairment with antidepressant use OR = 1.65 (95% CI: 1.18–2.31; *I^2^* = 35.2%) whereas those with participants aged less than 65 years (Cherbuin et al., 2009; Kessing et al., 2009) revealed an even stronger association with pooled OR = 3.25 (95% CI: 3.19–3.32; *I^2^* = 0%). Studies that factored in the use of other psychotropic medications (Angst et al., 2007; Cherbuin et al., 2009) had a pooled OR of 2.23 (95% CI: 1.14–4.45; *I^2^* = 58.8%) while those that did not (Goveas et al., 2012; Kessing et al., 2009; Sachdev et al., 2012), had an estimate of OR = 2.10 (95% CI: 1.17–3.77; *I^2^* = 89.7%)

The overall pooled estimate for all five studies was OR = 2.17 (95% CI: 1.41–3.33; *I^2^* = 84.9%), which was similar to the pooled estimate of OR = 2.00 (95% CI: 1.21–3.29) obtained when the study with the greatest effect estimate (Cherbuin et al., 2009) was excluded. Similarly, excluding the study with the largest sample size (Kessing et al., 2009), yielded a pooled estimate of OR = 1.85 (95% CI: 1.28–2.69). Influential analyses done using Tobias’ method (1999) showed that the pooled estimates obtained with the exclusion of one study at each turn always remained within the 95% CI of the overall estimates calculated for antidepressant use and some form of cognitive impairment. Thus, none of the studies had a significant individual, influential effect on the overall estimates.

Given the small number of studies included in this meta‐analysis, it was difficult to clearly ascertain the symmetry of the funnel plot. Thus, and in order to assess for publication bias, statistical testing for symmetry of the plot was completed using Begg's test (Begg & Mazumdar, 1994). The test was not statistically significant (adjusted Kendall's score = −4, *P* = .462) suggesting publication bias was unlikely. However, it should be noted that the statistical tests for funnel plot symmetry usually suffer from low power when the meta‐analysis includes a small number of studies (Sterne, Gavaghan, & Egger, 2000).

## 4 DISCUSSION

The prevalence of depression in the general population (World Health Organization, 2015b) has driven the prescription of antidepressant drugs, including SSRIs (Pratt et al., 2011). While these drugs do provide benefit in major depressive disorder, obsessive–compulsive disorder, and general anxiety disorder, there is evidence of adverse risk associated with most classes of antidepressant drugs (Kauffman, 2009). For example, the recent literature associates usage of antidepressant drugs, particularly SSRIs, with increased risk of outcomes as seemingly diverse as suicide (completion as well as attempts) (Braun, Bschor, & Franklin, 2016) and miscarriages (compared to risk in nondepressed women as well as depressed, but drug naïve women) (Almeida et al., 2016). Our pooled analysis now confirms that individuals with dementia/cognitive impairment are twice as likely to have been exposed to antidepressant drugs compared to those individuals without dementia/cognitive impairment. Data from four of the five studies included in our analysis individually demonstrate a similar association.

An important issue surrounding the interpretation of this association is confounding. Unadjusted estimates that are intended to quantify the effect of one variable on an outcome can be biased if the effects of more than one variable are intermixed. For example, an unadjusted OR describing the association of antidepressant and dementia/cognitive impairment may be biased upwards if depression itself is a risk factor for dementia/cognitive impairment and if depression is more common in those taking antidepressants, resulting in an intermixing of these effects. In pharmacoepidemiology, this type of confounding is referred to as confounding by indication (Neutel, 1993). We addressed this in our subanalysis by restricting study inclusion to studies in which all of the subjects are depressed. In such studies since all participants are depressed, the effect of depression cannot be intermixed with an effect of antidepressants such that confounding by indication cannot occur. However, confounding by severity is still a potential issue since those respondents taking antidepressants may be more severely depressed than those not taking antidepressants and a spurious association could arise through this mechanism.

Of all the subgroup analyses carried out, only the categorization by average age shows a significant difference, i.e. there is no overlap of the CI for the groups with an average age of 65 or older versus those less than age 65. Thus, age might be a significant interactor in the relationship between antidepressant drug usage and cognitive impairment. In both of these groups, there are increased odds noted, but the effect is more pronounced in those less than age 65. Though not statistically significant, there appears to be a stronger association between antidepressant drug use and dementia than that seen with antidepressant drug usage and mild cognitive impairment or any other type of cognitive impairment. As with the overall association, a stronger effect of antidepressants in older respondents may represent a causal effect of antidepressants on risk. However, other explanations cannot be ruled out. For example, if vascular disease in more elderly people leads more often to antidepressant prescriptions, subsequent worsening of cognition may be due to confounded effects of vascular disease and/or antidepressants.

The literature suggests an increasing burden of dementia with increasing age, most notably after age 65 (Richard et al., 2012; World Alzheimer Report, 2014). However, it is clear that AD/dementia, being a progressive, neurodegenerative disorder, likely develops from events, including depression, that are triggered well before the onset of any overt symptomatology (Emery, 2011; Green et al., 2003; Saczynski et al., 2010). Given the inevitable polypharmacy and long‐term antidepressant drug usage in any younger cohort with an early‐onset or persistent form of depression, a higher risk for off‐target biochemical effects of the drug(s)—with, for example, potential interactions with, or long‐term effects on, normal, age‐dependent metabolic, epigenetic and/or hormonal systems—and eventual iatrogenic cognitive phenotypes would not be unexpected. In keeping with this notion, a family history of an affective disorder can increase the likelihood of a depressive episode during the course of AD (Pearlson et al., 1990; Strauss & Ogrocki, 1996) and a younger age at onset of AD is associated with a greater depression (Lawlor et al., 1994), with depression worsening as AD progresses (Mega, Cummings, Fiorello, & Gornbein, 1996). It is clear that an effect of earlier age of onset of use may reflect longer use of an antidepressant or it may reflect a longer history of depression, so the issue of confounding remains a limitation here.

A decrease in serotonin availability within the synapse—a core feature of the monoamine theory of depression—can be reestablished by inhibiting monoamine oxidase (MAO, degrades serotonin and other monoamines) or by inhibiting serotonin reuptake/removal from the synapse. Depressed women tend to respond better to MAO inhibitors, while men tend to respond better to reuptake inhibitors (Davidson & Pelton, 1986). Interestingly, doses of SSRIs lower than the smallest available doses might still provide benefit, while reducing side effects and risk (Kauffman, 2009). In contrast, patient noncompliance and an effective “less than daily” dose could actually precipitate dementia and dementia‐related mortality (Rosness et al., 2016). It should be noted that AD/dementia is not associated with all classes of reuptake inhibitors. For instance, the older, tricyclic antidepressants, some of which function as serotonin uptake inhibitors, may be associated with a reduced risk (Lee et al., 2016) or no risk (Kessing et al., 2009) of dementia, whereas non‐SSRI antidepressant drugs, i.e. including MAO inhibitors and serotonin noradrenaline reuptake inhibitors (SNRIs), have been reported to show an intermediate risk (Kessing et al., 2009; Lee et al., 2016; Wang et al., 2015). These tentative findings suggest that the treatment of the depression rather than depression itself may have a powerful impact on cognitive phenotypes.

Similar patterns can be found in the relevant animal literature. For example, acute SSRI regimens have been associated with neuroprotective profiles on pathological markers as well as cognition and memory in mouse models of AD‐like neuropathology (discussed in (Sierksma et al., 2016)), whereas a chronic SSRI regimen is associated with seizures and increased mortality, as observed in males of an AD‐related mouse model (Sierksma et al., 2016). The latter observation is consistent with our findings of a higher, albeit modest, effect estimate in the study with male predominance of participants (current study) as well as our previous report of a higher risk of mortality in demented male patients with a comorbid psychiatric diagnosis (Meng et al., 2012).

Mouse models also indicate an age‐dependent shift in toxicity profile associated with antidepressant drugs. Indeed, studies involving the Down syndrome Ts65DN mouse model (note that Down syndrome and AD share several neuropathological markers and cognitive phenotypes, including dementia and depression) indicate that the SSRI fluoxetine can restore the depletion of serotonin, which appears to prevent the loss of dendritic arborization (Guidi et al., 2013) and the diminished neurogenesis in hippocampus (Bianchi et al., 2010; Clark et al., 2006), and, in turn, mitigates cognitive deficits (Bianchi et al., 2010) in these mice. However, any benefit of SSRI/fluoxetine in these mice is lost with age, given that fluoxetine exacerbates neuropathological phenotypes in older mice (Heinen et al., 2012).

Finally, as stated above, most cases of AD/dementia are influenced by factors such as advancing age (65+ years), biological sex and whether one carries the ε4 allele of the *APOE* gene (the only genetic risk unquestionably associated with the late‐onset form of AD). Interestingly, the *APOE* ε4 status is also associated with clinical depression, but only in women (Muller‐Thomsen et al., 2002), and particularly those with a history of depression prior to developing AD (Delano‐Wood et al., 2008). The interaction of ε4 genotype and depression further increases the risk of incident dementia (Geda et al., 2006). It would be interesting to revisit some of these larger population study datasets—for example, the extensive dataset associated with the Danish study (Meng et al., 2012)—to determine whether biological sex, *APOE* ε4 genotype (if described in the patient files) and specific antidepressant drug usage (including for off‐label indications (Wong et al., 2016)) interact to impart greater odds of developing a dementia‐related phenotype.

Limitations of note include the small pool of included studies, the presence of clinical and statistical heterogeneity, and the fact that some of the studies did not look at this association as their primary hypothesis. Thus, adjustments were not commonly made for other explanatory variables and therefore, it was difficult to confidently exclude the effect of within study confounding. This latter issue is particularly worrisome in pharmacoepidemiology, where confounding by indication and severity pose serious methodological challenges. Often, questions in pharmacoepidemiology can be addressed by meta‐regression, but such an analysis would normally require approximately 10 studies per determinant of heterogeneity. The paucity of relevant studies in the literature precludes any such study design. The best way to deal with the issue of confounding would be randomization, which, again, would rely on a greater number of studies. Often (when randomized studies are not feasible) advanced methods such as propensity score analysis are necessary to deal with these forms of bias. This is not possible in a meta‐analysis such as this one, in which only unadjusted estimates were consistently available for pooling. An association, in itself, does not confirm causation, but a strong association that is consistently observed, in which temporality of the association is clear and which is biologically plausible may represent an etiologic effect (Hill, 1965). The existence of an association, as confirmed here, creates an urgent need to explore the underlying issue of etiology.

## 5 CONCLUSION

In conclusion, our meta‐analysis suggests that individuals with cognitive impairment or AD/dementia are more likely to have been prescribed an antidepressant drug and that this is more evident if antidepressant drug treatment began before the age of 65. This increased association is strong, is biologically plausible, and is consistent across the various studies examined. The risk‐benefit trade‐offs of antidepressant treatment cannot be fully understood until the etiological basis of this association is more fully elucidated.
